# Pre-Transplant Hypoalbuminemia Is Not Associated With Early Key Outcomes Among Simultaneous Pancreas and Kidney Transplant Recipients

**DOI:** 10.3389/ti.2025.14091

**Published:** 2025-01-20

**Authors:** Ekaterina Fedorova, Sofia Nehring Firmino, Dixon B. Kaufman, Jon S. Odorico, David Aufhauser, Carrie Thiessen, David P. Al-Adra, Didier Mandelbrot, Brad C. Astor, Sandesh Parajuli

**Affiliations:** ^1^ Division of Transplant Surgery, Department of Surgery, University of Wisconsin School of Medicine and Public Health, Madison, WI, United States; ^2^ University of Wisconsin-Madison School of Medicine and Public Health, Madison, WI, United States; ^3^ Division of Nephrology, Department of Medicine, University of Wisconsin School of Medicine and Public Health, Madison, WI, United States; ^4^ Department of Population Health Sciences, University of Wisconsin School of Medicine and Public Health, Madison, WI, United States

**Keywords:** hypoalbuminemia, SPK, DGF, patient survival, graft survival

## Abstract

The role of pre-transplant hypoalbuminemia and its impact on post-transplant outcomes in patients undergoing simultaneous pancreas-kidney (SPK) transplantation remains unclear. We analyzed all SPK recipients at our center, who had at least 2 weeks of pancreas and kidney graft survival and had serum albumin measured within 45 days pre-transplant. Recipients were categorized based on pretransplant albumin level as normal (≥4.0 g/dL, N = 222, 42%), mild hypoalbuminemia (≥3.5–<4.0 g/dL, N = 190, 36%), and moderate hypoalbuminemia (<3.5 g/dL, N = 120, 23%). Kidney delayed graft function (DGF), length of stay (LOS) after transplant, re-hospitalization within 30 days after discharge, and need for a return to the operating room (OR) related to transplant surgical complications, acute rejection, and uncensored and death-censored graft failure, within the first years post-transplant were outcomes of interest. A total of 532 SPK recipients were included. Mild or moderate hypoalbuminemia was not associated with DGF, LOS, re-hospitalization, or return to the OR in unadjusted or adjusted analyses. Similarly, mild or moderate hypoalbuminemia was not associated with a risk of graft rejection or graft failure. Among SPK recipients, pre-transplant hypoalbuminemia was not associated with worse outcomes and should not be the determining factor in selecting patients for SPK transplant.

## Graphical Abstract

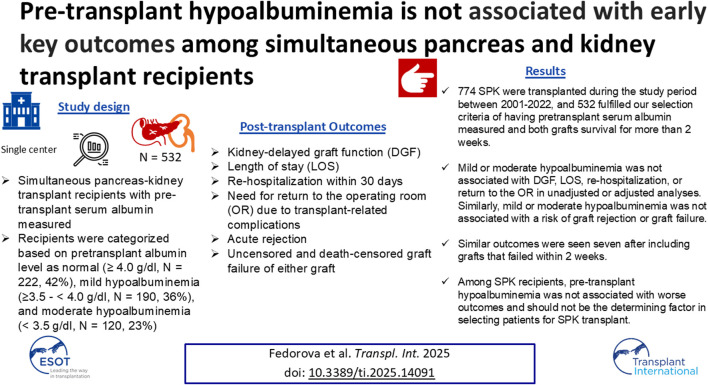

## Introduction

Albumin is the most abundant plasma protein in the human body and has many functions, including maintaining plasma colloid osmotic pressure, acting as a transport protein, buffering antioxidants, and having anticoagulant effects [1]. Hepatic synthesis of albumin depends on nutritional status and insulin stimulation; it can be decreased due to inflammatory cytokines, such as IL-6 and tumor necrosis factor-alpha [1, 2].

Hypoalbuminemia is multifactorial and results in increased capillary permeability and leakage. It may be related to the release of inflammatory cytokines, decreased nutritional intake, increased excretion, and altered rate of synthesis and catabolism of albumin [1–3]. Hypoalbuminemia is commonly seen in critically ill patients and is often associated with worse outcomes for a variety of medical and surgical conditions [4–9]. Hypoalbuminemia is associated with increased cardiovascular mortality as well as all-cause mortality in patients with end-stage renal disease (ESRD) undergoing hemodialysis [4]. Additionally, pre-operative hypoalbuminemia is associated with increased surgical morbidity, mortality, and increased risk of surgical complications [5–7]. In kidney-only transplantation, patients with pre-transplant hypoalbuminemia have an increased risk of BK and cytomegalovirus (CMV) infection [10]. However, pre-transplant hypoalbuminemia in kidney-only transplant recipients is not associated with an increased rate of re-admission, reoperation, or delayed graft function [11].

Simultaneous pancreas-kidney transplant (SPK) is a form of treatment for those with diabetes mellitus and ESRD as it restores euglycemia and slows the progression of diabetes complications [12, 13]. In these patients, hypoalbuminemia has been proposed to be caused by chronic systemic inflammation and reduced synthesis of albumin [14]. Additionally, post-operative hypoalbuminemia in SPK recipients is associated with an increased rate of CMV virus infection, graft loss, and a trend toward decreased survival [15]. However, the relationship between the severity of pre-transplant hypoalbuminemia and post-transplant outcomes among SPK recipients has not been thoroughly studied. We hypothesize that pre-transplant hypoalbuminemia is associated with an increased rate of postoperative complications in SPK recipients.

## Materials and Methods

### Study Population and Design

This single-center study from the University of Wisconsin-Madison included all adult SPK transplant recipients performed between 01/01/2001 and 12/31/2022. We excluded all recipients whose pancreas or kidney graft failed within 2 weeks of transplant, multiorgan transplant recipients (i.e., simultaneous liver - pancreas), or pancreas after kidney transplant recipients. In subgroup analysis, we analyzed SPK recipients even with graft failure within 2 weeks of transplant. This study was approved by the University of Wisconsin Institutional Review Board (IRB protocol number: 2014-1072). This study followed the Declaration of Helsinki. The clinical and research activities being reported were consistent with the Principles of the Declaration of Istanbul as outlined in “The Declaration of Istanbul on Organ Trafficking and Transplant Tourism.”

Only recipients with serum albumin levels measured within 45 days before transplant were included. In patients with multiple albumin levels measured within 45 days before transplant, we used the measurement closest to the transplant date. SPK recipients without albumin levels within the 45-day timeframe before transplant were excluded. Recipients were categorized based on pretransplant albumin levels as normal (≥4.0 g/dL), mild hypoalbuminemia (≥3.5–< 4.0 g/dL), and moderate hypoalbuminemia (<3.5 g/dL). Although, we initially planned to categorize the severe hypoalbuminemia group defined as <3.0 g/dL, this was not possible due to the extremely small sample size in this group with limited outcomes of interest, so for this reason they were included in the moderate hypoalbuminemia group. Pre or peri-transplant albumin transfusion to correct hypoalbuminemia has never been the current or past practice at our center, although few recipients may have received albumin infusion intraoperative or post-operative for various other indications. At no time during this study period or currently in our program was there a protocolized criterion for pretransplant serum albumin level to approve or disapprove SPK transplant eligibility.

Length of stay (LOS) after index transplant, kidney delayed graft function (DGF), and re-hospitalization within 30 days after discharge were perioperative outcomes of interest followed by the need for a return to the operating room (OR) related to transplant surgical complications, acute rejection of either grafts and uncensored and death-censored graft failure, within the 12 months post-transplant were short-term outcomes of interest. We limited outcomes to the first 12 months post-transplant to better correlate pre-transplant serum albumin levels and early post-transplant outcomes.

### Variables and Definitions

Donor characteristics such as age, sex, race, body mass index (BMI), death due to cardiovascular causes, terminal serum creatinine, kidney donor profile index (KPDI), donation after circulatory death (DCD), pancreas cold ischemia time (CIT), and kidney CIT were reported. Information on recipients included age, sex, race, BMI, diabetes type, induction therapy, pre-emptive transplant, and early steroid withdrawal. Immunologic factors reported were panel reactive antibody (cPRA) > 20%, average Human Leukocyte Antigen (HLA) mismatch (of 6), and whether primary or prior transplant.

DGF was defined as a need for dialysis within one-week post-transplantation. Re-hospitalizations within 30 days post initial discharge and any unanticipated return to the operating room within 12 months post-SPK were included. Return to the operating/procedure room for an anticipated ureteric stent removal was not considered a return to the OR. All episodes of rejections were biopsy-proven within 12 months of transplant. Kidney uncensored graft failure was defined as all causes of graft failure including death, while death-censored graft failure (DCGF) was defined as a return to dialysis or re-transplantation, all within 2 weeks–12 months post-transplant. Similarly, pancreas uncensored graft failure was defined as all causes of pancreas graft failure including death. And pancreas DCGF was defined based on the current United Network for Organ Sharing criteria for pancreas graft failure, which include removal of the pancreas graft, re-registration for a pancreas transplant, registration for an islet transplant after receiving pancreas, or an insulin requirement that is ≥0.5 units/kg/day for 90 consecutive days [16, 17].

### Serum Albumin Measurement

At our institution, serum albumin concentration measurements are conducted utilizing bromocresol assays. Before 2008, albumin was measured using bromocresol green assays. Since 2008, our institution has used bromocresol purple to assess serum albumin concentrations.

Reference ranges were adjusted to reflect the methodology of each test; prior institutional comparison of both assays on patient samples showed an average negative 0.56-g/dL bias in the newer bromocresol purple assay. This difference was considered to be within allowable limits for general clinical use.

### Immunosuppressive Protocols

Our center-specific induction immunosuppression therapy was consistent throughout the study period, either a depleting agent (alemtuzumab or anti-thymocyte globulin) or a non-depleting agent (basiliximab) was utilized. Triple immunosuppression with tacrolimus, mycophenolic acid and prednisone taper was standard for all recipients. Few had early steroid withdrawal based on the immunological risk and patient request as previously described [18].

### Biopsy and Rejection Protocols

The two most common indications for kidney biopsy were an unexplained rise in creatinine and proteinuria or the development of *denovo* DSA against HLA as described before [19]. Similarly, the common indications for pancreas biopsy were an unexplained rise in pancreatic enzymes, development of *denovo* DSA, and unexplained hyperglycemia [20]. If possible, we attempt to perform both graft biopsies in the setting of dual graft dysfunction [20].

Similarly, the management of rejections was based on the severity and proximity from the transplant to the diagnosis of rejection as described before [19, 20]. Briefly, kidney T cell-mediated rejection (TCMR) was treated with steroid pulse plus/minus anti-thymocyte globulin. And antibody-mediated rejection (AMR) was treated with steroid pulse, intravenous immunoglobulin (IVIG), plus/minus rituximab, plus/minus plasmapheresis. Treatment of pancreas rejection was based on the type and severity of rejection and was graded by the Banff criteria. TCMR was treated with IV steroid pulse with or without anti-thymoglobulin 6–12 mg/kg in 4–10 divided doses, while mixed rejection was treated with steroids, anti-thymoglobulin, IVIG, and plasmapheresis. Early AMR was treated with steroids, IVIG, and plasmaphereis [20].

### Statistical Analysis

Continuous data were compared using Student’s *t-*test or the Wilcoxon rank-sum test, where appropriate. Categorical data were analyzed using Fisher’s exact test or chi-square test. *p-*Values ≤ 0.05 were considered statistically significant. Multivariable logistic regression and Cox proportional hazard models were used to analyze associations of the pre-transplant serum albumin levels with various outcomes of interest. Variables considered to be associated with outcomes of interest from baseline characteristics were included in adjusted models. Outcomes of interest were also analyzed by Kaplan-Meier survival analysis. Analyses were performed in Stata SE 18.0^1^.

## Results

774 SPK recipients were transplanted during the study period, 23 were excluded due to early graft failure or early patient death all within 2 weeks of transplant, and 219 did not have serum albumin levels measured during the timeframe and were excluded from the study. With this, a total of 532 SPK recipients fulfilled our selection criteria. The details of recipient and donor baseline characteristics are summarized in Table 1. Recipient’s and donor’s characters differed across albumin categories in multiple variables, including the donor’s cause of death, the proportion of DCD donors, pancreas cold ischemia time, the proportion of previous transplant recipients, recipient’s sex, types of diabetes, induction immunosuppression and proportion of pre-emptive transplant recipients.

**TABLE 1:** Baseline characteristics of participants.

	Variables	Overall (N = 532)	<3.5 (n = 120)	≥3.5–<4.0 (n = 190)	≥4.0 (n = 222)	P
Donor Factors	Mean age (yrs)	28.0 (12.2)	27.7 (12.6)	26.3 (11.0)	29.7 (12.8)	0.06
	Female (%)	38.0	40.8	33.7	40.1	0.88
	Non-white (%)	13.2	12.5	13.7	13.1	0.92
	Mean body mass index (kg/m^2^)	23.7 (4.3)	23.8 (4.0)	23.8 (4.2)	24.1 (4.5)	0.35
	Cause of death: Cardiovascular (%)	19.0	17.5	13.2	24.8	**0.01**
	Terminal serum Creatinine (mg/dL)	1.03 (0.89)	0.90 (0.50)	1.08 (1.05)	1.06 (0.91)	0.15
	Mean kidney donor profile index %	20.2 (16.5)	21.3 (17.8)	17.3 (15.4)	21.8 (16.2)	0.79
	Donation after circulatory death (%)	14.3	19.2	15.3	10.8	**0.03**
	Pancreas Cold ischemia time (hrs)	14.5 (4.5)	13.2 (4.2)	14.5 (4.0)	15.2 (4.8)	**<0.001**
	Kidney Cold ischemia time (hrs)	15.3 (4.4)	14.8 (4.3)	15.2 (3.9)	15.6 (4.9)	0.14
Immunologic Factors	cPRA >20% (%)	14.2	11.5	17.9	12.2	0.95
	Mean HLA mismatch (of 6)	4.3 (1.2)	4.5 (1.2)	4.3 (1.3)	4.3 (1.2)	0.23
	Previous transplant (%)	16.9	6.7	18.4	21.1	0.001
Recipients Factors	Means pre-transplant serum albumin level	3.8 (0.6)	3.0 (0.4)	3.7 (0.1)	4.3 (0.3)	**<0.001**
	Mean age (yrs)	43.2 (9.3)	43.3 (9.8)	44.3 (9.7)	42.2 (8.7)	0.17
	Female (%)	39.7	47.5	41.6	33.8	**0.01**
	Non-white (%)	9.0	14.2	8.4	6.8	**0.03**
	Mean body mass index (kg/m^2^)	25.9 (3.9)	25.2 (3.9)	26.4 (4.0)	25.7 (3.9)	0.48
	Diabetes type					
	Type I	89.8	85.0	57.9	94.1	**0.02**
	Type II/Other	10.2	15.0	12.1	5.9	
	Induction Immunosuppression (%)					
	Alemtuzumab	35.5	24.2	32.1	44.6	**0.003**
	Anti-thymocyte globulin	24.3	30.0	26.3	19.4	
	Basiliximab	40.2	45.8	41.6	36.0	
	Early steroid withdrawal (%)	3.6	4.2	3.7	3.2	0.62
	Pre-emptive transplant	44.5	28.2	45.7	52.4	<0.001

Bold signifies statistical sigificant with p < 0.05.

### Kidney Delayed Graft Function

A total of 7.3% of recipients had DGF (Table 2). 4.5% of recipients with normal albumin levels, 9% with mild, and 10% with moderate hypoalbuminemia group experienced DGF. Mild hypoalbuminemia (OR: 2.08; 95% CI: 0.93–4.67; p 0.07) and moderate hypoalbuminemia were not associated with risk of DGF (OR: 2.36; 95% CI: 0.99–5.63; p = 0.05) in an unadjusted model. This was further confirmed after adjustment of multiple variables in mild (OR: 1.69; 95% CI: 0.65–4.37; p = 0.28); moderate (OR: 1.52; 95% CI: 0.55–4.20; p = 0.42) hypoalbuminemia groups (Table 3).

**TABLE 2:** Complications, DGF, LOS, rehospitalization, Re-operation.

Complications		DGF, %	LOS, mean (sd)	Rehospitalization, %	Re-operation, %
	Pre-Tx albumin (g/dL)				
	≥4.0	4.5	11.2 (6.5)	32.4	25.7
	≥3.5–<4.0	9.0	10.4 (6.0)	35.3	20.0
	<3.5	10.0	12.1 (12.0)	35.8	20.8
	Overall	7.3	11.1 (7.9)	34.2	22.6

**TABLE 3:** Delayed graft function (n = 39).

Complications	Pre-Tx albumin	Unadjusted			Adjusted		
		OR	95% CI	p-value	OR	95% CI	p-value
DGF	≥4.0	Ref	Ref	Ref	Ref	Ref	Ref
	≥3.5–<4.0	2.08	0.93, 4.67	0.07	1.69	0.65, 4.37	0.28
	≥3.0–<3.5	2.36	0.99, 5.63	0.051	1.52	0.55, 4.20	0.42

Adjusted for age, sex, race, diabetes type, pre-emptive transplant, induction immunosuppression, pancreas cold time, donor age.

### Length of Stay

The mean LOS among the entire cohort after index transplant was 11.1 ± 7.9 days (Table 2). The mean LOS in normal, mild, and moderate hypoalbuminemia recipients was 11.2 ± 6.5 days, 10.4 ± 6.0 days, and 12.1 ± 12.0 days, respectively. Mild hypoalbuminemia was not associated with increased or decreased LOS in unadjusted (OR: −0.78; 95% CI: −2.31, 0.76; p = 0. 32) or adjusted models (OR: −0.94; 95% CI: −2.49, 0.61; p = 0. 24). Moderate hypoalbuminemia was also not associated with LOS in unadjusted (OR: 0.92; 95% CI: −0.84, 2.61; p = 0. 31) or adjusted models (OR: 0.22; 95% CI: −1.61, 2.06; p = 0. 81) (Table 4).

**TABLE 4:** Length of stay, mean LOS.

Complications	Pre-Tx albumin	Unadjusted			Adjusted		
		Coef	95% CI	p-value	Coef	95% CI	p-value
Length of stay	≥4.0	Ref	Ref	Ref	Ref	Ref	Ref
	≥3.5–<4.0	−0.78	−2.31, 0.76	0.32	−0.94	−2.49, 0.61	0.24
	<3.5	0.92	−0.84, 2.61	0.31	0.22	−1.61, 2.06	0.81

Adjusted for age, sex, race, diabetes type, pre-emptive transplant, induction immunosuppression, pancreas cold time, donor age.

### Rehospitalization

A total of 34.2% were re-hospitalizations within 30 days after the initial discharge from the index transplant (Table 2). The rate of rehospitalization in normal, mild, and moderate hypoalbuminemia recipients were 32.4%, 35.3%, and 35.8% respectively. Mild hypoalbuminemia was not associated with re-hospitalization rates in unadjusted (OR: 1.13; 95% CI: 0.75–1.71; p = 0.55) or adjusted models (OR: 1.31; 95% CI: 0.86–2.82; p = 0.21). Similarly, moderate hypoalbuminemia was not associated with re-hospitalization rates in unadjusted (OR: 1. 16; 95% CI: 0.73–1.86; p = 0.53) or adjusted models (OR: 1. 34; 95% CI: 0.81–2.23; p = 0. 25) (Table 5). The most common indications for re-hospitalization were gastrointestinal symptoms including, nausea, vomiting, diarrhea, and abdominal pain followed by hypo or hypertension.

**TABLE 5:** Re-hospitalization within 30 days after initial discharge (n = 182).

Complications	Pre-Tx albumin	Unadjusted			Adjusted		
		OR	95% CI	p-value	OR	95% CI	p-value
Re-hospitalization	≥4.0	Ref	Ref	Ref	Ref	Ref	Ref
	≥3.5–<4.0	1.13	0.75, 1.71	0.55	1.31	0.86, 2.82	0.21
	<3.5	1.16	0.73, 1.86	0.53	1.34	0.81, 2.23	0.25

Adjusted for age, sex, race, diabetes type, pre-emptive transplant, induction immunosuppression, pancreas cold time, donor age.

### Reoperations Within 2 Weeks–12 Months

**FIGURE 1:**
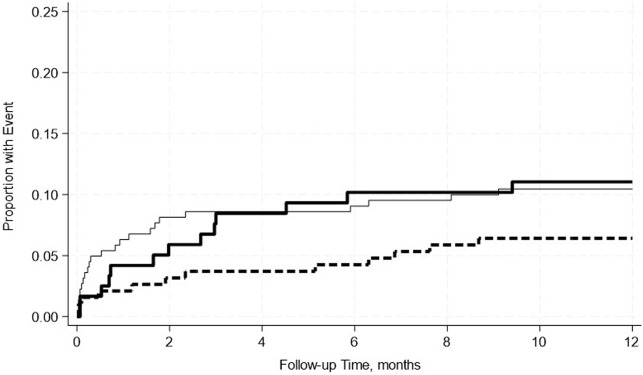
No significant differences in the incidence rate ratio of re-operation within 2 weeks–12 months post-transplant by serum albumin levels. The thin solid line represents the normal pre-transplant serum albumin levels group, the thick dashed line represents the mild hypoalbuminemia group and the thick solid line represents the moderate hypoalbuminemia group.

A total of 22.6% of recipients returned to the operating room within 2 weeks–12 months post-transplant (Table 2). 25.7% of patients with normal albumin levels, 20% with mild, and 20.8% with moderate hypoalbuminemia were reoperated within the stated timeframe. Mild hypoalbuminemia was not associated with increased reoperation rates in unadjusted (OR: 0.59; 95% CI: 0.29–1.18; p = 0.14) or adjusted models (OR: 0.54; 95% CI: 0.26, 1.12; p = 0.10). Similarly, moderate hypoalbuminemia was not associated with increased reoperation rates in unadjusted (OR: 1.04; 95% CI: 0.52–2.04; p = 0.92) or adjusted models (OR: 0.80; 95% CI: 0.38–1.66; p = 0. 54) (Table 6). This was further confirmed by the Kaplan-Meier survival analysis curve (Figure 1). The most common indications for return to the operating room were intraabdominal fluid collection followed by intrabdominal bleeding.

**TABLE 6:** Need to go to OR related to transplant within 12 months (n = 48).

Complications	Pre-Tx albumin	Unadjusted			Adjusted		
		HR	95% CI	p-value	HR	95% CI	p-value
Re-operation	≥4.0	Ref	Ref	Ref	Ref	Ref	Ref
	≥3.5–<4.0	0.59	0.29, 1.18	0.14	0.54	0.26, 1.12	0.10
	<3.5	1.04	0.52, 2.04	0.92	0.80	0.38, 1.66	0.54

Adjusted for age, sex, race, diabetes type, pre-emptive transplant, induction immunosuppression, pancreas cold time, donor age.

### Acute Rejection Within 2 Weeks–12 Months

**FIGURE 2:**
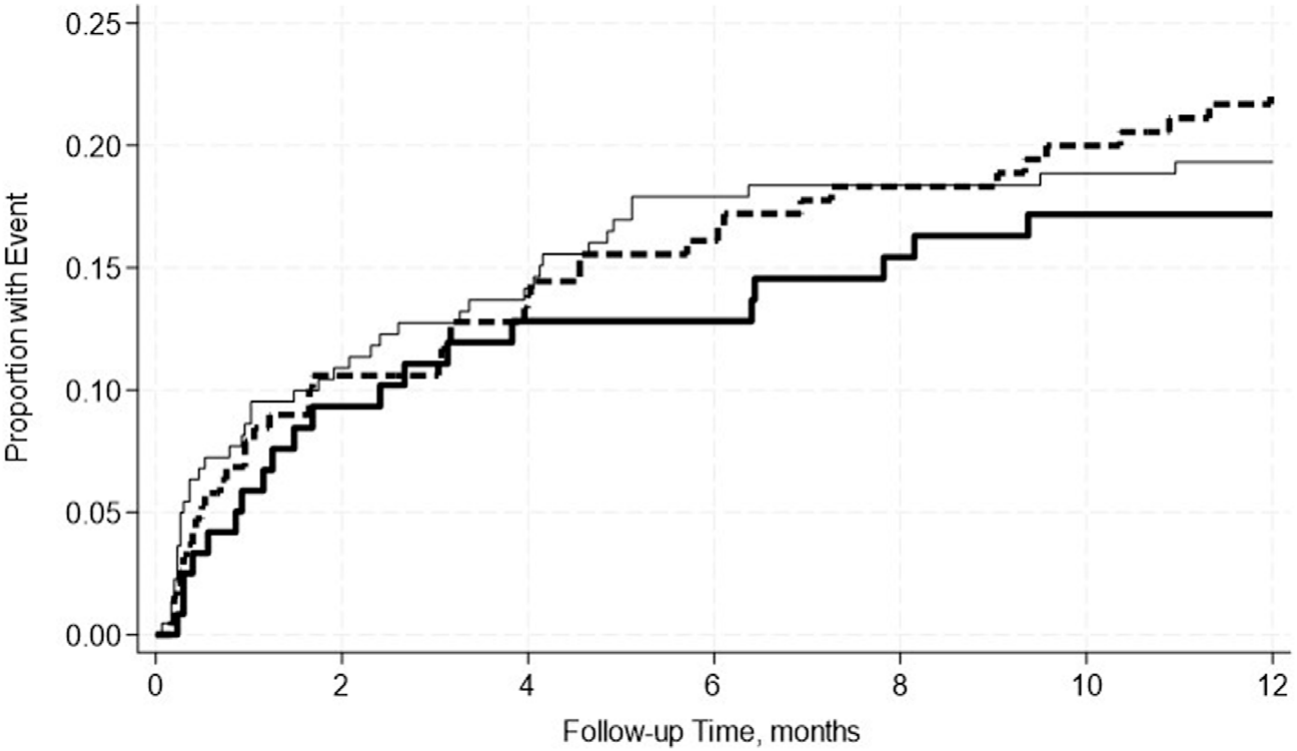
No significant differences in the incidence rate ratio of pancreas rejection within 2 weeks–12 months post-transplant by serum albumin levels. The thin solid line represents the normal pre-transplant serum albumin levels group, the thick dashed line represents the mild hypoalbuminemia group and the thick solid line represents the moderate hypoalbuminemia group.

**FIGURE 3:**
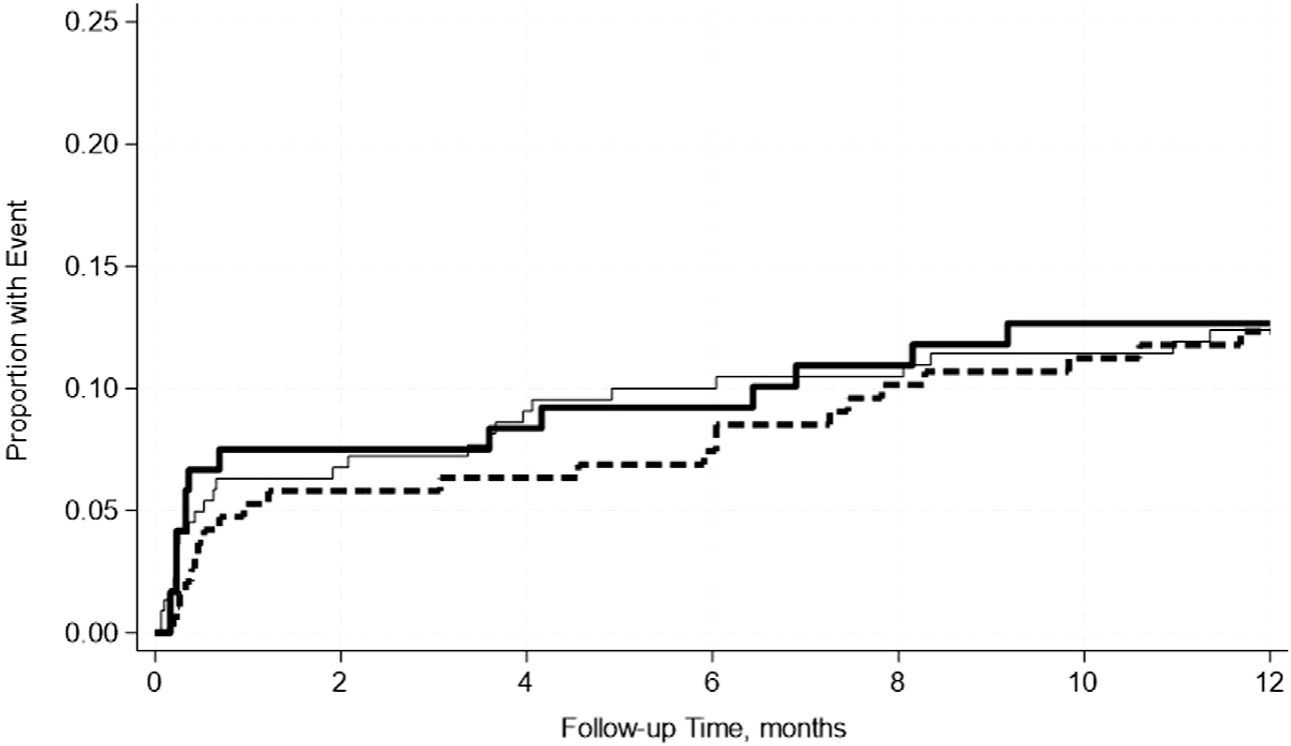
No significant differences in the incidence rate ratio of kidney rejection within 2 weeks–12 months post-transplant by serum albumin levels. The thin solid line represents the normal pre-transplant serum albumin levels group, the thick dashed line represents the mild hypoalbuminemia group and the thick solid line represents the moderate hypoalbuminemia group.

A total of 19.4% of SPK recipients developed pancreas rejection and 12.2% developed kidney rejection. Pre-transplant hypoalbuminemia, either mild or moderate was not associated with acute rejection in either adjusted or unadjusted models (Table 7). This was further confirmed by the Kaplan-Meier Survival analysis curve (Figures 2, 3).

**TABLE 7:** Acute rejection within 12 months.

Complications	Pre-Tx albumin	Unadjusted			Adjusted		
		HR	95% CI	p-value	HR	95% CI	p-value
Pancreas acute rejection (n = 103)	≥4.0	Ref	Ref	Ref	Ref	Ref	Ref
	≥3.5–<4.0	1.13	0.74, 1.74	0.57	1.16	0.75, 1.71	0.51
	<3.5	0.86	0.51, 1.47	0.58	0.81	0.46, 1.41	0.45
Kidney acute rejection (n = 65)	≥4.0	Ref	Ref	Ref	Ref	Ref	Ref
	≥3.5–<4.0	0.97	0.56, 1.70	0.92	0.98	0.55, 1.73	0.94
	<3.5	1.02	0.55, 1.93	0.94	0.81	0.41, 1.59	0.54

### Graft Failures Within 2 Weeks–12 Months

**FIGURE 4:**
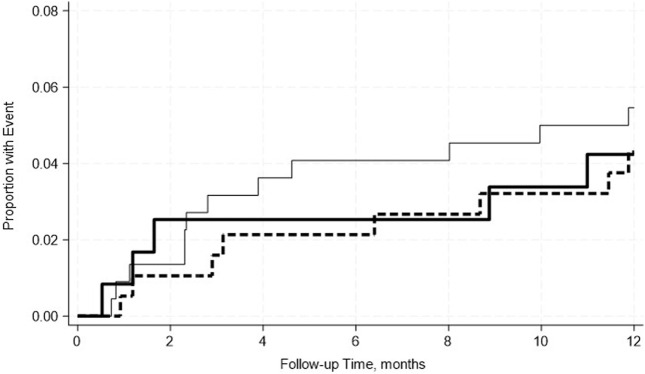
No significant differences in the incidence rate ratio of the pancreas death censored graft failure within 2 weeks–12 months post-transplant by serum albumin levels. The thin solid line represents the normal pre-transplant serum albumin levels group, the thick dashed line represents the mild hypoalbuminemia group, and the thick solid line represents the moderate hypoalbuminemia group.

**FIGURE 5:**
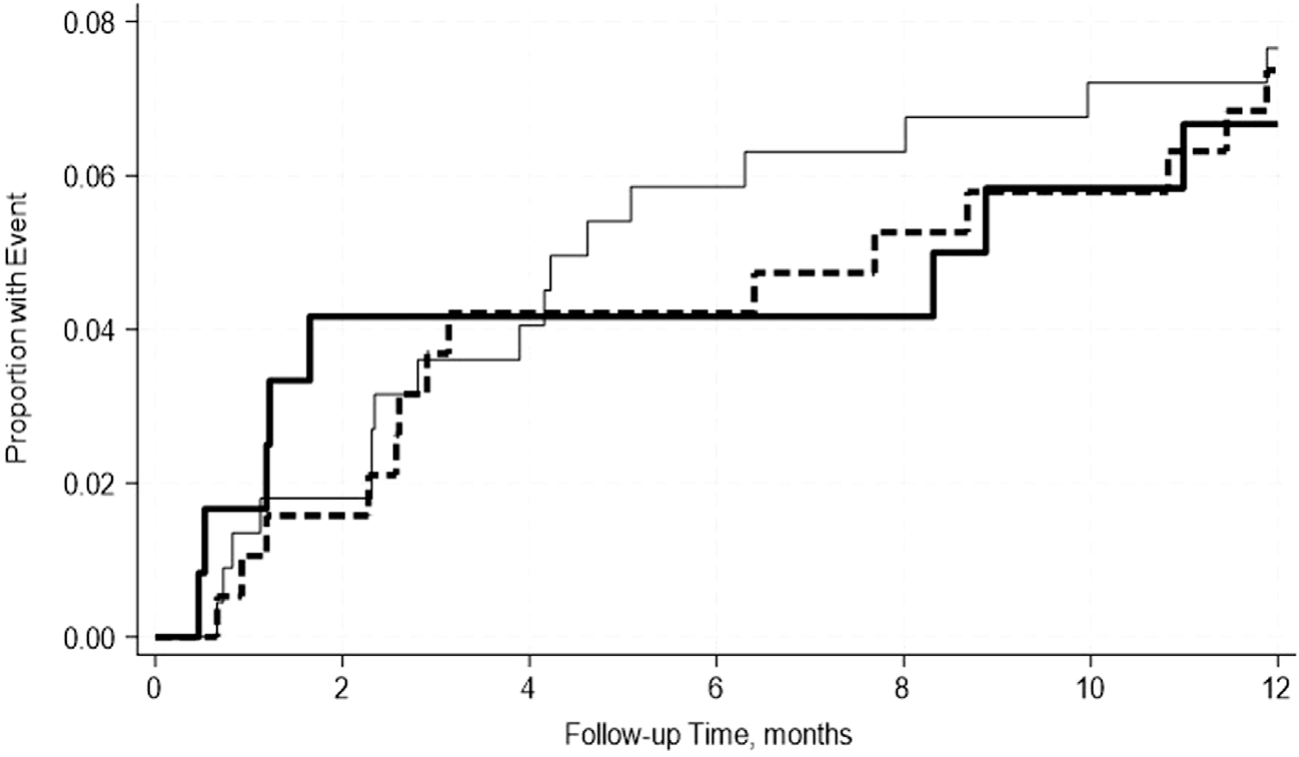
No significant differences in the incidence rate ratio of the pancreas uncensored graft failure within 2 weeks–12 months post-transplant by serum albumin levels. The thin solid line represents the normal pre-transplant serum albumin levels group, the thick dashed line represents the mild hypoalbuminemia group, and the thick solid line represents the moderate hypoalbuminemia group.

**FIGURE 6:**
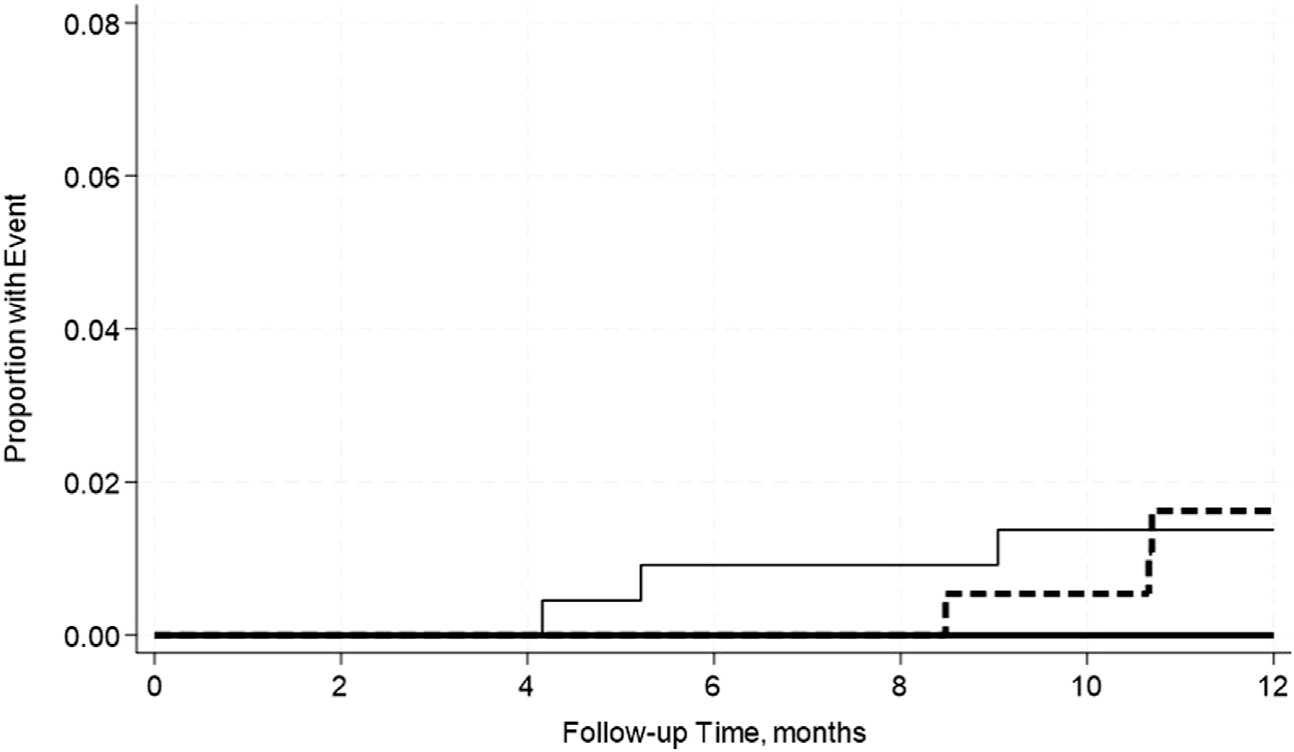
No significant differences in the incidence rate ratio of kidney death censored graft failure within 2 weeks–12 months post-transplant by serum albumin levels. The thin solid line represents the normal pre-transplant serum albumin levels group, the thick dashed line represents the mild hypoalbuminemia group and the thick solid line represents the moderate hypoalbuminemia group.

**FIGURE 7:**
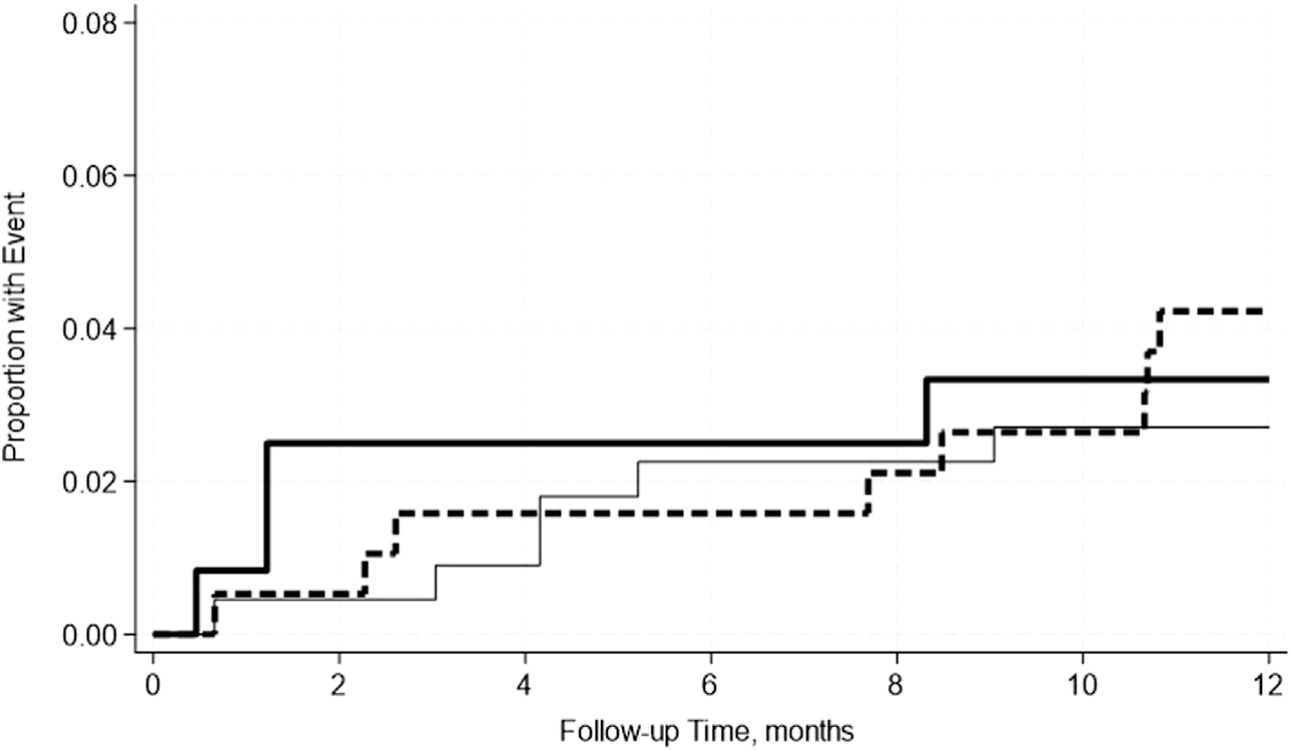
No significant differences in the incidence rate ratio of kidney uncensored graft failure within 2 weeks–12 months post-transplant by serum albumin levels. The thin solid line represents the normal pre-transplant serum albumin levels group, the thick dashed line represents the mild hypoalbuminemia group and the thick solid line represents the moderate hypoalbuminemia group.

A total of 7.3% of SPK recipients experienced uncensored graft failure and 4.7% experienced pancreas DCGF. Similarly, 3.3% experienced kidney uncensored graft failure, and 1.1% experienced kidney DCGF (Table 8). Pre-transplant hypoalbuminemia either mild or moderate was not associated with either uncensored or death-censored graft failure in either adjusted or unadjusted models. This was further confirmed by the Kaplan-Meier Survival analysis curve (Figures 4–7).

**TABLE 8:** Graft failure within 12 months.

Complications	Pre-Tx albumin	Unadjusted			Adjusted		
		HR	95% CI	p-value	HR	95% CI	p-value
Uncensored pancreas graft failure (n = 39)	≥4.0	Ref	Ref	Ref	Ref	Ref	Ref
	≥3.5–<4.0	0.96	0.47, 1.94	0.90	1.10	0.53, 2.31	0.80
	<3.5	0.87	0.38, 2.02	0.75	0.86	0.35, 2.11	0.75
Death censored pancreas graft failure (n = 25)	≥4.0	Ref	Ref	Ref	Ref	Ref	Ref
	≥3.5–<4.0	0.77	0.32, 1.90	0.58	0.97	0.38, 2.49	0.96
	<3.5	0.77	0.27, 2.19	0.63	0.79	0.26, 2.40	0.68
Uncensored kidney graft failure (n = 18)	≥4.0	Ref	Ref	Ref	Ref	Ref	Ref
	≥3.5–<4.0	1.56	0.54, 4.50	0.41	1.52	0.51, 4.57	0.45
	<3.5	1.24	0.35, 4.40	0.74	1.12	0.29, 4.27	0.87
Death censored kidney graft failure (n = 6)	≥4.0	Ref	Ref	Ref	Ref	Ref	Ref
	≥3.5–<4.0	1.17	0.24, 5.79	0.85	X	x	X
	<3.5	x	X	x	x	x	X
Death with functioning graft (n = 16)	≥4.0	Ref	Ref	Ref	Ref	Ref	Ref
	≥3.5–<4.0	1.16	0.37, 3.59	0.80	1.20	0.37, 3.91	0.77
	<3.5	1.24	0.34, 4.39	0.74	1.14	0.30, 4.35	0.84

Also, 3.0% of SPK recipients experienced death with both functional grafts, and similar to the previous findings, neither mild nor moderate pre-transplant hypoalbuminemia was associated with death with both functional grafts (Table 8).

A total of 17 SPK recipients had early pancreas DCGF, 3 had early kidney DCGF, and 3 died with both functional grafts, all within 2 weeks post-transplant, and were not included in the main analysis of the study. When analyzing the data in this subgroup of SPK recipients, pretransplant hypoalbuminemia was not associated with those outcomes of interest either in the mild or moderate hypoalbuminemia group in the unadjusted model (Supplementary Table S1).

Also when including these recipients with early pancreas graft failure, pretransplant hypoalbuminemia was not associated with either grafts uncensored or DCGF or death either in the mild or moderate hypoalbuminemia group in the unadjusted model or adjusted model (Supplementary Table S2).

## Discussion

In this large cohort of 532 SPK recipients, we found that pre-transplant hypoalbuminemia was not significantly associated with any detrimental early post-transplant outcomes. We looked for various common early post-transplant outcomes and events including kidney DGF, length of stay, readmission, re-operation, and graft failure, and none of these feared outcomes were associated with pre-transplant hypoalbuminemia.

Hypoalbuminemia is a known risk factor for various detrimental outcomes in patients who suffer from diabetes mellitus and ESRD. Inflammation has been identified to play a key role in the pathogenesis of hypoalbuminemia in this group of patients. Chronic systemic inflammation that suppresses both innate and acquired immunity ultimately places patients with concomitate diabetes and ESRD at higher risk of life-threatening infections, morbidity, and death [4, 21, 22]. Therefore, we expected to observe a negative effect of hypoalbuminemia on both patient and graft survival. Breyer et al., also did not observe worse outcomes among kidney transplant recipients, although they report a lower risk of acute rejection based on the degree of hypoalbuminemia [11].

Patients with diabetes and ESRD are at higher risk for malnutrition [23]. Among solid organ transplant recipients, malnutrition has been associated with various poor clinical outcomes among liver, kidney, heart, and lung transplant recipients [23, 24].

In the past, serum albumin was thought to be an acceptable sole marker of nutritional status but has since been widely researched and refuted [23]. A retrospective cohort study from 2006 found no association between serum albumin and mortality risk among lung transplant recipients but found a higher risk of death in recipients with a low prealbumin level (<18 g/dL) [25]. Still, pre-albumin and albumin levels should not be considered valid tools for malnutrition diagnosis, as they are influenced by multiple factors including inflammation and fluid status. According to Evans et al, albumin and prealbumin as acute phase proteins do not consistently or predictably change with weight loss, calorie restriction, or nitrogen balance and more accurately indicate the severity of the inflammatory response rather than poor nutrition status [26]. In October 2020, the American Society for Parenteral and Enteral Nutrition (ASPEN) published a position paper stating that albumin and prealbumin are not components of any accepted definitions of malnutrition [26]. Extrapolating from previous data among various solid organ transplants, even among SPK recipients, our results support that serum albumin levels should not be used to assess nutritional status in this unique population. Although, out of the scope of this study, in the future, if an association between malnutrition and outcomes among SPK recipients is to be sought, should include various other nutritional tools and should not solely rely on the serum albumin levels.

To the best of our knowledge, there is no other study assessing the risk of pre-transplant hypoalbuminemia in SPK recipients with various post-transplant outcomes. In one study, Becker et al reported that persistent post-SPK hypoalbuminemia was associated with an increased risk of CMV infection, both kidney and pancreas graft failure, and a trend toward increased risk of overall patient mortality [15]. In another study, among kidney-only transplant recipients, Anderson et al. reported that hypoalbuminemia was an independent risk factor for overall graft failure after kidney transplantation [27]. Furthermore, several other studies showed that kidney transplant recipients with hypoalbuminemia were at increased risk for all-cause mortality, cardiovascular mortality, graft failure, DGF, acute rejection, BK, and CMV infections [10, 28–31].

To the best of our knowledge, this is the first study that investigated mild and moderate hypoalbuminemia and its associations with numerous post-transplant outcomes among patients undergoing SPK. Data that originated from a single-center hospital study allowed us to provide nuanced, granular data points, that reflected a homogeneous approach to transplant practices involving both medical and surgical patient management. These unique characteristics are unavailable with large multicenter registry datasets. Nonetheless, our study had several limitations. We were unable to identify the exact causes of hypoalbuminemia. Additionally, it was out of the scope of this study to assess the risk of infections and malignancies based on the pre-transplant serum albumin levels. Also, we did not assess the outcomes based on the changes in serum albumin levels post transplant. Lastly, due to having stringent selection criteria for SPK, most of the recipients were likely to be in relatively good health, and despite having mild/moderate hypoalbuminemia they were able to recover from SPK transplantation no differently than those without hypoalbuminemia. Also, we did not assess the various pre-transplant risk factors that usually coexist with hypoalbuminemia including pre-transplant peritoneal dialysis modality, frailty, nutritional status, muscle mass, fluid overload etc.

To summarize, the outcomes of this study have significant clinical importance showing that various degrees of hypoalbuminemia, were not associated with inferior outcomes particularly when it comes to death-censored and death uncensored graft failure. We conclude that patients with mild or moderate hypoalbuminemia, as defined in this study, who are otherwise acceptable candidates for SPK, should be considered for transplantation. Undoubtedly, future research on this topic is necessary to address the limitations reported above. Also, research to identify some of the easily available biomarkers predicting various post-transplant outcomes in these populations will be beneficial.

### Summary

Simultaneous pancreas-kidney transplant (SPK) has become a growing form of treatment for those with diabetes mellitus and ESRD as it restores euglycemia and slows the progression of diabetes complications. In these patients, hypoalbuminemia has been proposed to be caused by chronic systemic inflammation and reduced synthesis of albumin. However, the role of pre-transplant hypoalbuminemia and its impact on post-transplant outcomes in patients undergoing SPK transplantation remains unclear. In this study, we studied 532 SPK recipients at our center with mild and moderate hypoalbuminemia. Outcomes of interest included kidney delayed graft function (DGF), length of stay (LOS) after transplant, re-hospitalization within 30 days after discharge, and need for a return to the operating room related to transplant surgical complications, acute rejection, and uncensored and death-censored graft failure, within the first years post-transplant. Mild or moderate hypoalbuminemia was not associated with DGF, LOS, re-hospitalization, return to the operating room, graft rejection, or graft failure. The outcomes of this study have significant clinical importance showing that mild or moderate hypoalbuminemia, was not associated with inferior outcomes, concluding that these patients are acceptable candidates for SPK.

## Data Availability

The raw data supporting the conclusions of this article will be made available by the authors, without undue reservation.

## Footnotes

1
http://www.stata.com

## Abbreviations

ABMR, Antibody-Mediated Rejection; aHR, Adjusted Hazard Ratio; BMI, Body Mass Index; CIT, Cold Ischemia Time; CMV, Cytomegalovirus; cPRA, Calculated Panel Reactive Antibody; DCGF, Death Censored Graft Failure; DCD, Donation After Circulatory Death; ESRD, End Stage Renal Disease; HLA, Human Leukocyte Antigen; KDPI, Kidney Donor Profile Index; KDGF, Kidney Delayed Graft Function; LOS, Length of Stay; SPK, Simultaneous Pancreas and Kidney; TCMR, T-cell Mediated Rejection.
