# Supplementary material for: Pre-Transplant Hypoalbuminemia Is Not Associated With Early Key Outcomes Among Simultaneous Pancreas and Kidney Transplant Recipients
Source: Transpl Int. 2025 Jan 20;38:14091. doi: 10.3389/ti.2025.14091 (PMC11789475; doi:10.3389/ti.2025.14091)
Supplement: Supplementary file 1 [file Table1.DOCX]

ST1: Associated with early graft failure within 2 weeks post transplant

|  |  | Unadjusted | | |
| --- | --- | --- | --- | --- |
| **Complications** | **Pre-Tx albumin** | **OR** | **95% CI** | **p-value** |
| Pancreas death censored graft failure  N=17 | ≥ 4.0 | Ref | Ref | Ref |
|  | ≥ 3.5 - <4.0 | 1.48 | 0.39, 5.62 | 0.56 |
|  | <3.5 | 1.86 | 0.60, 5.78 | 0.28 |
| Kidney death censored graft failure  N=3 | ≥ 4.0 | Ref | Ref | Ref |
|  | ≥ 3.5 - <4.0 | 5.60 | 0.58, 54.4 | 0.14 |
|  | <3.5 | 5.82 | 0.67, 50.3 | 0.17 |
| Patient death  N=3 | ≥ 4.0 | Ref | Ref | Ref |
|  | ≥ 3.5 - <4.0 | 3.70 | 0.33,41.3 | 0.29 |
|  | <3.5 | 3.46 | 0.36, 34.5 | 0.28 |
