# Supplementary material for: Pre-Transplant Hypoalbuminemia Is Not Associated With Early Key Outcomes Among Simultaneous Pancreas and Kidney Transplant Recipients
Source: Transpl Int. 2025 Jan 20;38:14091. doi: 10.3389/ti.2025.14091 (PMC11789475; doi:10.3389/ti.2025.14091)
Supplement: Supplementary file 2 [file Table2.DOCX]

ST2: Including all SPK recipients with pre-transplant albumin levels measured during the study period

|  |  | Unadjusted | | | Adjusted | | |
| --- | --- | --- | --- | --- | --- | --- | --- |
| Complications | Pre-Tx albumin | HR | 95% CI | p-value | HR | 95% CI | p-value |
| Uncensored pancreas graft failure (n=59) | ≥ 4.0 | Ref | Ref | Ref | Ref | Ref | Ref |
|  | ≥ 3.5 - <4.0 | 0.91 | 0.44, 1.88 | 0.81 | 0.95 | 0.44, 2.04 | 0.90 |
|  | <3.5 | 1.15 | 0.64, 2.09 | 0.63 | 1.36 | 0.74, 2.51 | 0.33 |
| Death censored pancreas graft failure (n=42) | ≥ 4.0 | Ref | Ref | Ref | Ref | Ref | Ref |
|  | ≥ 3.5 - <4.0 | 0.86 | 0.37, 1.99 | 0.73 | 0.93 | 0.38, 2.24 | 0.87 |
|  | <3.5 | 1.09 | 0.55, 2.15 | 0.81 | 1.35 | 0.67, 2.74 | 0.40 |
| Uncensored kidney graft failure (n=24) | ≥ 4.0 | Ref | Ref | Ref | Ref | Ref | Ref |
|  | ≥ 3.5 - <4.0 | 1.87 | 0.60, 5.79 | 0.28 | 1.59 | 0.47, 5.30 | 0.45 |
|  | <3.5 | 1.93 | 0.70, 5.30 | 0.20 | 1.85 | 0.65, 5.28 | 0.25 |
| Death censored kidney graft failure (n=9) | ≥ 4.0 | Ref | Ref | Ref | Ref | Ref | Ref |
|  | ≥ 3.5 - <4.0 | 1.24 | 0.21, 7.42 | 0.81 | 1.45 | 0.19, 10.83 | 0.72 |
|  | <3.5 | 1.92 | 0.46, 8.03 | 0.37 | 2.28 | 0.47, 10.97 | 0.31 |
| Death with functioning graft (n=19) | ≥ 4.0 | Ref | Ref | Ref | Ref | Ref | Ref |
|  | ≥ 3.5 - <4.0 | 1.07 | 0.31, 3.66 | 0.91 | 0.99 | 0.27, 3.59 | 0.99 |
|  | <3.5 | 1.32 | 0.48, 3.65 | 0.59 | 1.37 | 0.47, 3.94 | 0.56 |

Adjusted for age, sex, race, diabetes type, pre-emptive transplant, induction immunosuppression, pancreas cold time, donor age
